# A PRISMA-compliant meta-analysis of MDM4 genetic variants and cancer susceptibility

**DOI:** 10.18632/oncotarget.12558

**Published:** 2016-10-11

**Authors:** Yajing Zhai, Zhijun Dai, Hairong He, Fan Gao, Lihong Yang, Yalin Dong, Jun Lu

**Affiliations:** ^1^ Department of Pharmacy, The First Affiliated Hospital, Xi'an Jiaotong University, Xi'an, Shaanxi, 710061, China; ^2^ Department of Oncology, The Second Affiliated Hospital of Xi'an Jiaotong University, Xi'an, Shaanxi, 710061, China; ^3^ Clinical Research Center, The First Affiliated Hospital, Xi'an Jiaotong University, Xi'an, Shaanxi, 710061, China

**Keywords:** MDM4, polymorphism, cancer susceptibility, meta-analysis

## Abstract

Molecular epidemiological research suggests that mouse double minute 4 (MDM4) polymorphisms may be associated with cancer susceptibility, but results remain controversial. To derive a more precise evaluation, we performed a PRISMA compliant meta-analysis focused on five single nucleotide polymorphisms (rs11801299, rs1380576, rs10900598, rs1563828, and rs4245739) of MDM4. Overall, 23 studies involving 22,218 cases and 55,033 controls were analyzed. The results showed that rs4245739 was significantly associated with a decreased cancer risk in the allelic (C vs. A: odds ratio [OR] = 0.848, 95% confidence interval [CI] = 0.765–0.941, *P* = 0.002), heterozygous (AC vs. AA: OR = 0.831, 95% CI = 0.735–0.939, *P* = 0.003), and dominant (AC+CC vs. A: OR = 0.823, 95% CI = 0.727–0.932, *P* = 0.002) models. The association was more prominent in Asians. No significant association was found using any genetic model for the rs11801299, rs1380576, rs10900598, and rs1563828 SNPs. These results indicate that the rs4245739 polymorphism may contribute to a decreased cancer susceptibility and support the hypothesis that genetic variants in the MDM4 genes act as important modifiers of cancer risk.

## INTRODUCTION

Mouse double minute 4 (MDM4, also known as MDMX) is a member of the MDM family that also includes MDM2 and its derivatives. MDM4 can bind directly to p53 and inhibit its transcriptional activation, as well as mediate various cellular pathways based on p53 [1]. Tumor suppressor protein p53 plays an important role in regulating cell growth, division, and apoptosis. Overactive MDM4 may reduce p53 tumor suppression function and contributes to tumor formation and progression [2]. MDM4 was found to be up-regulated in invasive breast carcinoma (by 14.2%), liver hepatocellular carcinoma (by 12.4%), retinoblastomas (by 65%), skin cutaneous melanomas (by 12%), and stage II–V melanomas (by > 65%) [3].

Molecular epidemiological research suggests that genetic variations in MDM4 gene may be associated with the cancer risk. Recently, a single nucleotide polymorphism (SNP) in the 3′ untranslated region of the MDM4 gene, rs4245739 A > C has been found to affect MDM4 mRNA stability and protein levels [4]. Genotype AA was recorded to be more frequent in patients with high-grade than low-grade ovarian carcinoma [5]. Furthermore, several studies indicated the rs4245739 C allele to be associated with a reduced risk for non-Hodgkin lymphoma [6], breast cancer [7], esophageal squamous cell carcinoma [8], and prostate cancer [9]. In 2009, Atwal and colleagues found that specific SNPs in MDM4 (rs10900594, rs2290853, rs2369244, and rs12039454) may affect p53 tumor-suppression activity [10]. Moreover, the presence of these SNPs in Ashkenazi Jewish and European cohorts has been associated with increased risks of early-onset breast and ovarian cancers. In short, several SNPs in the MDM4 have been associated with elevated or reduced cancer risk, but data are at variance. We therefore performed the PRISMA- compliant meta-analysis of the accumulated information and evaluated the associations of MDM4 polymorphisms with cancer susceptibility.

## RESULTS

### Characteristics of included studies

**Figure 1:**
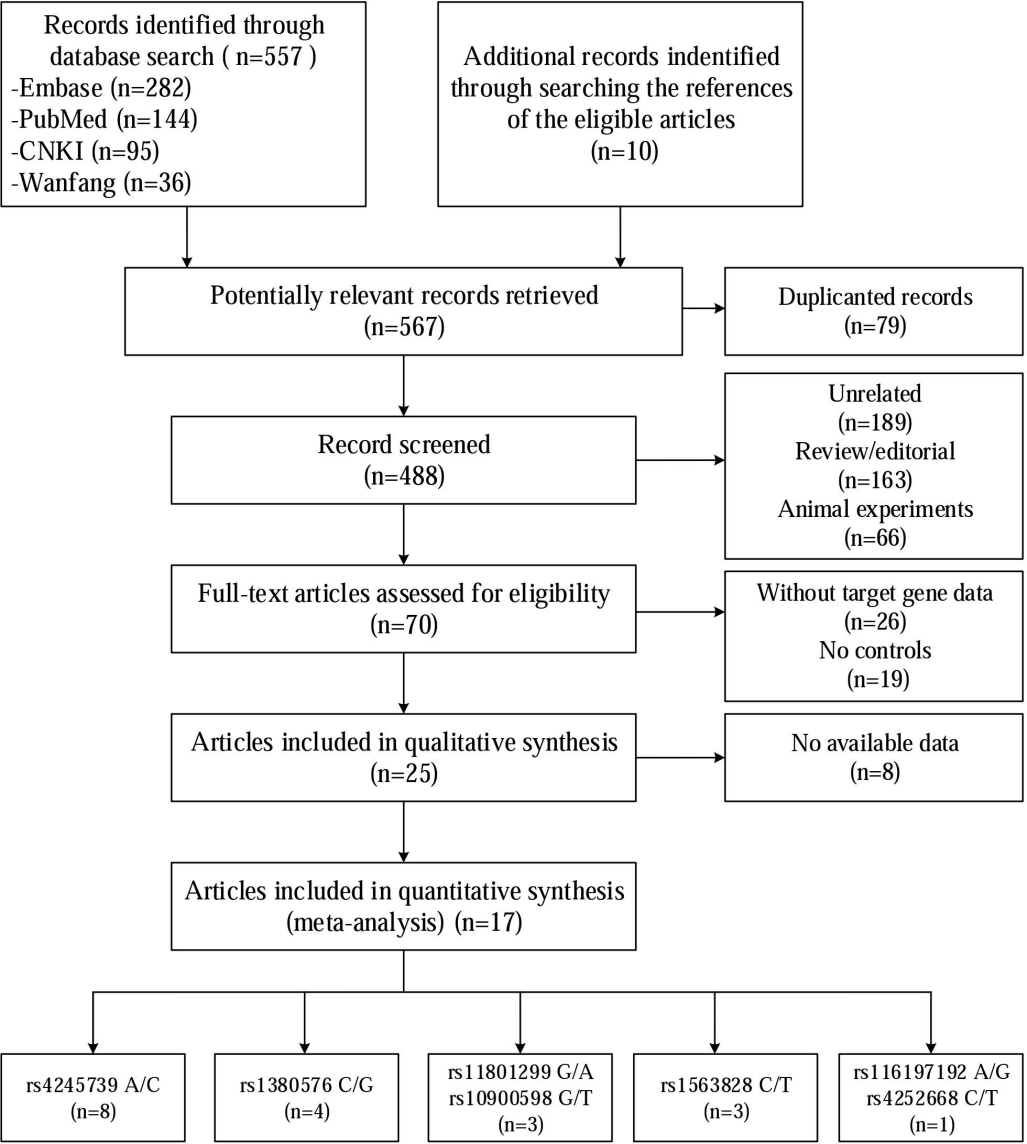
Selection of studies of association between MDM4 genetic variants and cancer susceptibility

Figure 1 shows a flow chart of the studies selection procedure. From 567 initial studies, 497 were discarded at title or abstract level. Another 45 studies did not meet the prespecified inclusion criteria and were therefore excluded. Of the remaining 25 articles, 8 articles were also excluded due to some data being unavailable even after contacting the corresponding authors. Ultimately, 17 articles focusing on the association between MDM4 polymorphisms and cancer risk were identified. Only one of the studies involved rs116197192 and rs4252668, so we did not include these two SNPs in the subsequent meta-analysis [11]. The remaining 16 articles involved the following 5 SNPs: rs11801299 [12–14], rs10900598 [12–14], rs1380576 [12–15], rs1563828 [16–18], and rs4245739 [5–8, 19–23]. Five of the articles described multiple case–control studies of different types of cancer or different populations. Overall, 23 eligible case–control comparisons that involved 22,218 cancer patients and 55,030 controls were enrolled in this meta-analysis, with 3 studies considered to be of low quality (quality score < 10) [5, 11, 16] and 20 were of high quality (quality score ≥ 10) [6–8, 12–15, 17–23]. Within the distribution of genotypes in the control groups, all studies are consistent with Hardy-Weinberg equilibrium (HWE). Table 1 presents the characteristics of the individual studies.

**Table 1:** Characteristics of studies in the meta-analysis

Author	Year	Ethnicity	Genotyping method	Cancer type	Case/control	Control source	HWE	Quality score (0–15)	Polymorphism site
Wynendaele [14]	2010	Caucasian	PCR-RFLP	OC	154/154	HB	*P* = 0.982	7	rs4245739
Yu [7]	2011	Caucasian	TaqMan	SCCHN	1075/1079	HB	*P* = 0.084 for rs11801299, *P* = 0.712 for rs1380576, *P* = 0.398 for rs10900598	11	rs11801299, rs1380576, rs10900598
Oliveira [6]	2012	Caucasian, Mullato, Black	PCR/RFLP	RB	104/104	PB	*P* = 0.683 for rs116197192, *P* = 0.802 for rs4252668	8	rs116197192, rs4252668
Song [11]	2012	Asian	MassArray	BC	124/101	HB	*P* = 0.862	7	rs1563828
Wang [8]	2012	Caucasian	TaqMan	Oral cancer	320/321	HB	Agreement with HWE*	11	rs11801299, rs1380576, rs10900598
Yu [9]	2012	Caucasian	TaqMan	SCCHN	380/335	HB	*P* = 0.303 for rs11801299, *P* = 0.502 for rs1380576, *P* = 0.669 for rs10900598	10	rs11801299, rs1380576, rs10900598
Zhang [12]	2012	Asian	RT-PCR	NPC	210/200	PB	*P* = 0.944	10	rs1563828
Garcia [22]	2013	Caucasian	Illumina array	BC	6512/41451	Mixed	*P* = 0.183	11	rs4245739
Liu [15]	2013	Asian	PCR-RFLP	BC	800/800 (Jinan); 300/600 (Huaian)	PB	*P* = 0.505 for Jinan, *P* = 0.483 for Huaian	13	rs4245739
Zhou [16]	2013	Asian	PCR-RFLP	ESCC	540/550 (Jinan); 588/600 (Huaian)	PB	*P* = 0.740 for Jinan, *P* = 0.379 for Huaian	13	rs4245739
Fan [17]	2014	Asian	PCR-RFLP	NHL	200/400	PB	*P* = 0.487	12	rs4245739
Thunell [13]	2014	Caucasian	TaqMan	HCM	50/799	PB	*P* = 0.725	11	rs1563828
Feng [18]	2014	Asian	PCR-RFLP	GN	419/494	HB	*P* = 0.561	10	rs4245739
Gansmo [19]	2015	Caucasian	LightSNiP assay	BC (*n* = 1,717); LC (*n* = 1,331); CC (*n* = 1,531); PC (*n* = 2,500)	7079/3747	PB	*P* = 0.566	13	rs4245739
Gao [20]	2015	Asian	PCR-RFLP	LC	320/640 (Jinan); 200/400 (Huaian)	PB	*P* = 0.399 for Jinan, *P* = 0248 for Huaian	10	rs4245739
Wu [10]	2015	Asian	TaqMan	GN	642/720	PB	*P* = 0.46	13	rs1380576
Gansmo [21]	2016	Caucasian	LightSNiP assay	EC (*n* = 1404); OC (*n* = 1385)	2789/1870	PB	*P* = 0.106	13	rs4245739

* This study only offered dominant-model data for rs11801299, rs1380576, and rs10900598, thus the p value of HWE were not calculated.

BC: breast cancer, CC: colon cancer, EC: endometrial cancer, ESCC: esophageal squamous cell carcinoma, GN: gastric neoplasms, HB: hospital based, HCM: hereditary cutaneous melanoma, LC: lung cancer, NHL: non-Hodgkin lymphoma, NPC: nasopharyngeal carcinoma, OC: ovarian carcinomas, PB: population based, PC: prostate cancer, RB: retinoblastoma, RT-PCR: reverse transcription-PCR, SCCHN: squamous cell carcinoma of the head and neck, SCLC: small cell lung cancer

### Quantitative analysis

**Figure 2:**
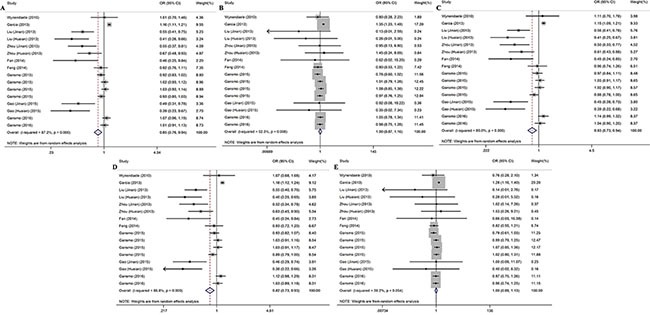
Forest plots for the association between the rs4245739 polymorphisms and cancer risk under five genetic models (**A**) allelic model; (**B**) homozygous model; (**C**) heterozygous model; (**D**) dominant model; (**E**) recessive model.

Table 2 presents the main results of this meta-analysis. The 16 studies of rs4245739 found that this SNP was significantly associated with a decreased cancer risk in the allelic (C vs. A: odds ratio [OR] = 0.848, 95% confidence interval [CI] = 0.765–0.941, *P* = 0.002), heterozygous (AC vs. AA: OR = 0.831, 95% CI = 0.735– 0.939, *P* = 0.003), and dominant (AC + CC vs. A: OR = 0.823, 95% CI = 0.727–0.932, *P* = 0.002) models [5–8, 19–23]. However, the relationship remains controversial in the other genetic models (Figure 2).

**Table 2:** The result of meta-analysis for various genotype models

SNP	Covariates	Variables	No. of studies	Sample size (case/control)	Allele		Homozygous		Heterozygous		Dominant		Recessive	
					OR (95%CI)	*P^h^/I^2^*	OR (95%CI)	*P^h^/I^2^*	OR (95%CI)	*P^h^/I^2^*	OR (95%CI)	*P^h^/I^2^*	OR (95%CI)	P^h^/I^2^
rs4245739 A/C		All	16	19950/49914	0.848 (0.765-0.941)	<0.001/87.2%	1.002 (0.866-1.159)	0.008/52.3%	0.831 (0.735-0.939)	<0.001/85.0%	0.823 (0.727-0.932)	<0.001/86.8%	0.981 (0.394-2.444)	<0.001/99.1%
	Ethnicity	Asian	8	3416/4483	0.561 (0.439-0.718)	0.001/71.1%	0.782 (0.536-1.141)	0.910/0.0%	0.547 (0.428-0.698)	0.007/64.0%	0.544 (0.428-0.692)	0.007/64.1%	0.806 (0.562-1.156)	0.918/0.0%
		Caucasian	8	16534/45431	1.022 (0.948-1.101)	<0.001/78.6%	1.028 (0.875-1.208)	0.001/71.7%	1.037 (0.960-1.119)	0.060/64.3%	1.034 (0.949-1.126)	<0.001/74.3%	1.304(0.394-4.316)	<0.001/99.6%
	Cancer type	BC	3	9329/44721	0.766 (0.573-1.025)	<0.001/94.3%	0.937 (0.545-1.608)	<0.001/83.3%	0.776 (0.571-1.055)	<0.001/92.1%	0.759 (0.547-1.054)	<0.001/93.7%	0.945 (0.590-1.512)	0.002/79.3%
		LC	3	1128/1150	0.607 (0.308-1.194)	<0.001/90.4%	1.075 (0.844-1.370)	0.762/0.0%	0.584 (0.288-1.185)	<0.001/90.0%	0.585 (0.839-1.068)	<0.001/90.5%	1.067 (0.843-1.352)	0.814/0.0%
		ESCC	2	1851/4787	0.616 (0.478-0.793)	0.438/0.0%	1.197 (0.318-4.500)	0.759/0.0%	0.565 (0.431-0.741)	0.484/0.0%	0.580 (0.445-0.757)	0.464/0.0%	1.273 (0.339-4.785)	0.763/0.0%
		OC	2	1539/2023	1.065 (0.958-1.183)	0.767/0.0%	1.009 (0.778-1.308)	0.641/0.0%	1.141 (0.993-1.312)	0.917/0.0%	1.119 (0.980-1.278)	0.841/0.0%	0.954 (0.741-1.229)	0.653/0.0%
		Other	6	6103/8314	0.960 (0.878-1.049)	0.080/52.1%	0.964 (0.841-1.105)	0.917/0.0%	0.949 (0.835-1.079)	0.044/59.1%	0.947 (0.839-1.068)	0.049/57.9%	0.972 (0.851-1.110)	0.912/0.0%
	Source of controls	HB	2	622/646	0.935 (0.790-1.106)	0.648/0.0%	0.803 (0.547-1.180)	0.985/0.0%	0.999 (0.791-1.262)	0.598/0.0%	0.960 (0.769-1.199)	0.588/0.0%	0.812 (0.563-1.170)	0.895 /0.0%
		PB	13	12816/18898	0.803 (0.714-0.903)	<0.001/83.0%	0.969 (0.874-1.075)	0.869/0.0%	0.768 (0.664-0.890)	<0.001/83.4%	0.768 (0.667-0.886)	<0.001/83.6%	0.967 (0.874-1.069)	0.906 /0.0%
		Mixed	1	6512/41451	1.159 (1.112-1.207)	–	1.355 (1.229-1.494)	–	1.148 (1.087-1.213)	–	1.180 (1.120-1.243)	–	1.277 (1.162-1.405)	–
rs11801299 G/A		All (Caucasian)	2	1446/1400	1.715 (0.531-5.545)	<0.001/98.5%	3.549 (0.302-41.765)	<0.001/97.2%	1.583 (0.523-4.794)	<0.001/97.1%	1.816 (0.471-7.003)	<0.001/98.2%	2.817 (0.401-19.803)	<0.001/95.6%
rs1380576 C/G		All	3	2088/2120	1.018 (0.931-1.114)	0.393/0.0%	1.002 (0.831-1.208)	0.558/0.0%	1.094 (0.958-1.250)	0.918/0.0%	1.070 (0.944-1.212)	0.771/0.0%	0.943 (0.797-1.116)	0.461/0.0%
	Ethnicity	Asian	1	642/720	0.939 (0.807-1.091)	–	0.895 (0.676-1.184)	–	1.093 (0.846-1.411)	–	1.008 (0.798-1.273)	–	0.849 (0.671-1.075)	–
		Caucasian	2	1446/1400	1.065 (0.953-1.191)	0.735/0.0%	1.098 (0.853-1.413)	0.850/0.0%	1.095 (0.937-1.279)	0.680/0.0%	1.096 (0.945-1.270)	0.681/0.0%	1.052 (0.827-1.339)	0.948/0.0%
rs10900598 G/T		All (Caucasian)	2	1446/1400	0.530 (0.163-1.729)	<0.001/98.7%	0.253 (0.019-3.363)	<0.001/98.2%	0.559 (0.193-1.615)	<0.001/96.6%	0.481 (0.126-1.832)	<0.001/98.1%	0.341 (0.043-2.713)	<0.001/97.4%
rs1563828 C/T		All	3	384/1100	0.909 (0.738-1.120)	0.750/0.0%	0.768 (0.474-1.244)	0.642/0.0%	0.972 (0.728-1.297)	0.867/0.0%	0.929 (0.706-1.223)	0.825/0.0%	0.776 (0.490-1.229)	0.657/0.0%
	Ethnicity	Asian	2	334/301	0.948 (0.750-1.199)	0.928/0.0%	0.859 (0.510-1.445)	0.921/0.0%	0.999 (0.718-1.392)	0.678/0.0%	0.969 (0.708-1.325)	0.764/0.0%	0.855 (0.522-1.402)	0.804/0.0%
		Caucasian	1	50/799	0.776 (0.488-1.236)	–	0.412 (0.096-1.770)	–	0.890 (0.494-1.603)	–	0.808 (0.456-1.434)	–	0.434 (0.103-1.823)	–
	Cancer type (Source of controls)	BC (HB)	1	124/101	0.962(0.651-1.421)	–	0.830(0.358-1.926)	–	1.100(0.627-1.932)	–	1.034(0.608-1.757)	–	0.791(0.358-1.747)	–
		Other (PB)	2	260/999	0.889(0.695-1.138)	0.493/0.0%	0.740(0.411-1.335)	0.350/0.0%	0.930(0.664-1.301)	0.859/0.0%	0.893(0.647-1.232)	0.680/0.0%	0.768(0.437-1.352)	0.358/0.0%

Seven studies examined the associations of the other four SNPs (rs11801299, rs10900598, rs1380576, and rs1563828) with the risk of cancer [12–18], but no significant associations were found (Table 2 and Supplementary Table S2).

### Meta regression

The *Q* statistic and the point estimate (*I*^2^) indicated the presence of high heterogeneity between studies in the meta-analysis of rs4245739, rs11801299, and rs10900598 (i.e., *P* < 0.10 and/or *I*^2^ > 50%).

For the meta-analysis of rs4245739, which involved more than 10 studies, we performed a meta-regression to determine the potential source of heterogeneity. Table 3 indicates that the main sources of significant heterogeneity were ethnicity (*P* < 0.001) and genotyping methods (*P* < 0.010).

**Table 3:** The results of meta-regression for rs4245739

Covariates	Number of dummy variables^a^	C vs. A	CC vs. AA	AC vs. AA	AC + CC vs. AA	CC vs. AC + AA
Publication year	–	0.518	0.279	0.536	0.536	0.133
Ethnicity	2	< 0.001	0.238	< 0.001	< 0.001	0.281
Cancer type	5	0.166	0.065	0.086	0.119	0.061
Genotyping methods	3	0.006	0.001	0.008	0.004	0.002
Source of controls	3	0.204	–	0.229	0.200	0.002

a The Bonferroni correction was used according the number of dummy variables. The statistical significance level that should be used for each covariate separately is 0.050, 0.025, 0.010, 0.017, and 0.017 respectively.

### Subgroup analysis

The subgroup analysis stratified by ethnicity indicated that rs4245739 decreased the risk of cancer in the allelic (C vs. A: OR = 0.561, 95% CI = 0.439–0.718, *P* < 0.001), heterozygous (AC vs. AA: OR = 0.547, 95% CI = 0.428–0.698, *P* < 0.001), and dominant (AC+CC vs. A: OR = 0.544, 95% CI = 0.428–0.692, *P* < 0.001) models in Asian but not Caucasian populations. Similarly, significant correlations with reduced cancer risk were also observed with three genetic models in population-based control groups (C vs. A: OR = 0.803, 95% CI = 0.714–0.903, *P* < 0.001; AC vs. AA: OR = 0.768, 95% CI = 0.664–0.890, *P* < 0.001; AC + CC vs. A: OR = 0.768, 95% CI = 0.667–0.886, *P* < 0.001). Moreover, reduced risks of esophageal squamous cell carcinoma was detected (Table 2).

Subgroup analyses were also performed for the other four SNPs, but no significant association was found (Table 2).

### Sensitivity analysis

**Figure 3:**
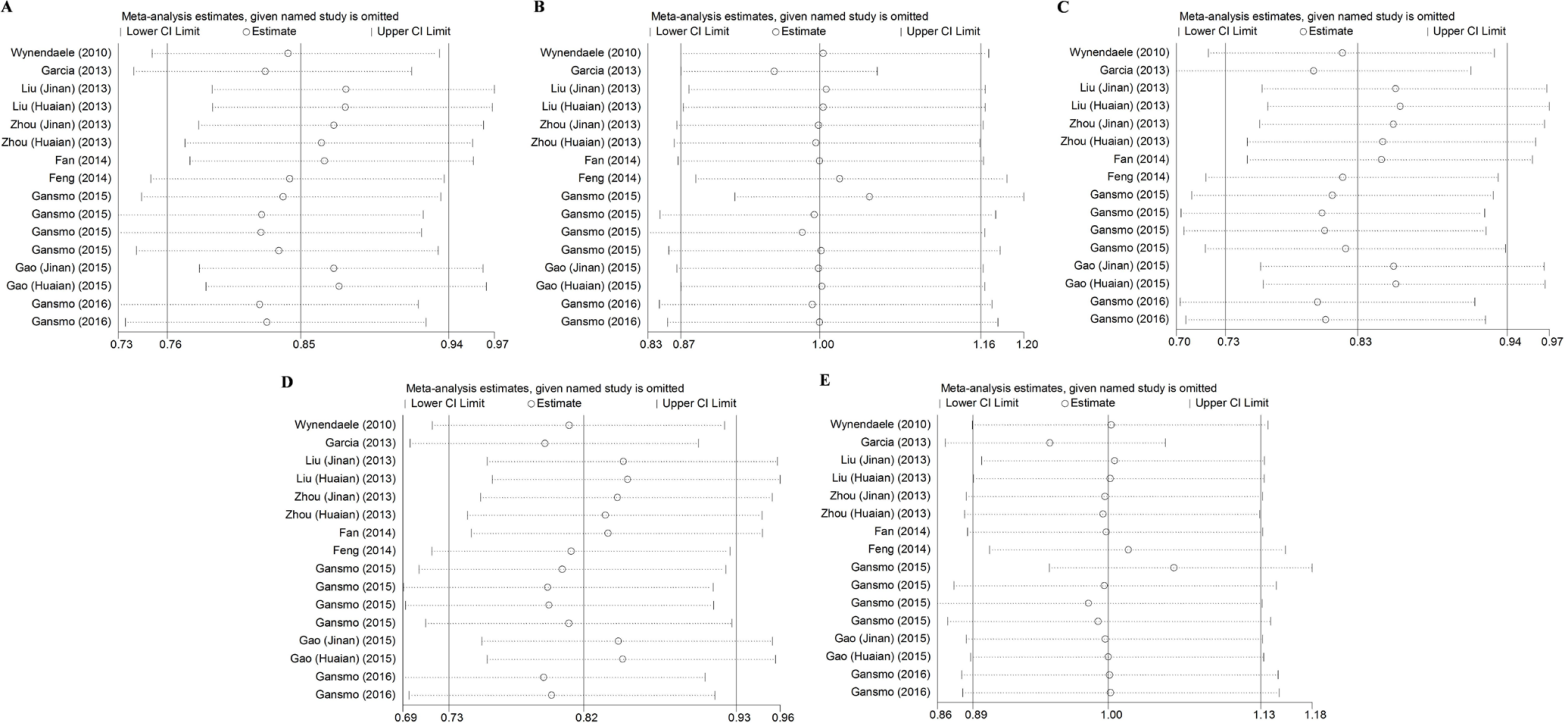
Leave-one-out analysis of association between the rs4245739 polymorphisms and cancer risk under five genetic models (**A**) allelic model; (**B**) homozygous model; (**C**) heterozygous model; (**D**) dominant model; (**E**) recessive model.

The results of the leave-one-out analysis of rs4245739 indicated that no individual study excessively influenced the pooled effect in any genetic models of the meta-analysis (Figure 3). Removing the three studies [5, 11, 16] considered to be of low quality from the meta-analysis of rs4245739 and rs1563828 did not change the results significantly (Supplementary Table S3). For rs11801299, rs1380576, and rs10900598, we added a study [13] that only produced dominant-model data, but no conspicuous change in the pooled ORs was detected (Supplementary Table S3).

### Publication bias

**Figure 4:**
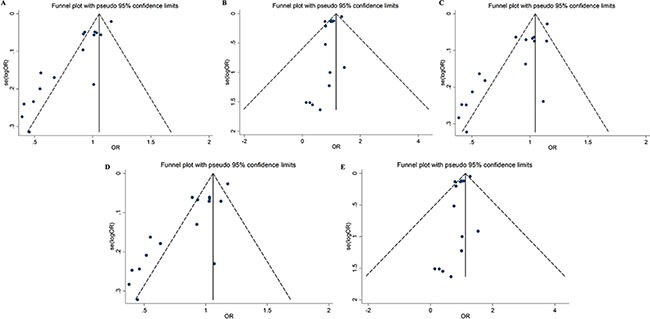
Funnel plots for the association between the rs4245739 polymorphisms and cancer risk under five genetic models (**A**) allelic model; (**B**) homozygous model; (**C**) heterozygous model; (**D**) dominant model; (**E**) recessive model.

Figure 4 shows funnel plots for the meta-analysis of rs4245739. The funnel plots are asymmetrical and Egger's test indicated the presence of significant publication bias in five genetic models (Egger's test: *P* < 0.001 for allele, heterozygous and dominant models; *P* = 0.010 for homozygous model; *P* = 0.011 for recessive model). The results did not change after the correction using the trim and fill method. We also calculated the fail-safe numbers for the positive results, which were 80 for the allelic, heterozygous, and dominant genetic models. This suggests there would need to be 80 unpublished studies to render the findings of the meta-analysis nonsignificant.

Publication bias was not tested for the meta-analysis of the other SNPs due to the small number of included studies (i.e., less than five each).

## DISCUSSION

How genetic factors influence the susceptibility of patients to cancer is receiving increasing attention [24, 25]. Prompted by the important role of MDM4 in the development of cancer, we have conducted the first comprehensive meta-analysis of the relationships between MDM4 polymorphisms and the risk of cancer. The results showed that the MDM4 rs4245739 polymorphism is associated with a significantly decreased risk of cancer. A subgroup analysis by ethnicity revealed that carriers of the C allele and mutated genotypes had a significantly lower cancer risk than Asian wild-type carriers, suggesting that the decreased cancer risk is ethno-specific. In addition, the other SNPs of MDM4 analyzed (i.e., rs11801299, rs1380576, rs10900598, and rs1563828) were not found to be associated with the risk of cancer.

The MDM4 gene is located on chromosome 1q32, which is an important regulator of the p53 pathway *in vivo* [2]. Elevated expression of MDM4 has been seen in both relatively rare (e.g., retinoblastoma and ocular melanoma) and more common (e.g., cutaneous melanoma and breast cancer) types of tumor [26]. There is also an increasing recognition that MDM4 is a promising and relatively safe therapeutic target for p53 reactivation therapy.

SNPs are the most common variations in genetic sequences and can alter the splicing of primary transcripts or gene expression, and may further affect the susceptibility, progression, and prognosis of diseases [27]. Functional SNPs have been identified in the MDM4 gene since 2009. Rs2279744 was reported under positive evolutionary selection to be associated with the risk of breast and ovarian cancers and with human fertility in Caucasian populations [10, 28], while rs1563828 was found to be associated with an earlier age at the diagnosis of estrogen-receptor-negative but not estrogen-receptor-positive breast cancers[29]. In the last decades, rs4245739 A > C was widely studied, which was found to create a functional target site for hsa-miR-191 and hsa-miR-887. Both miRs bind to the C-allele with higher affinity than to the A-allele, leading to miR-mediated decrease in MDM4 protein levels in cells carrying the C variant [4, 5]. Several case–control studies assessing this mutation in various cancer forms (esophageal squamous cell carcinoma, non-Hodgkin lymphoma, breast cancer, and prostate cancer), have found the C-allele to be associated with reduced risk, but have all been performed in Chinese populations [6–9]. Notably, there is a substantial difference in the distribution of this SNP between Europeans and Asians with a MAF of 0.26 and 0.05, respectively [30]. The somewhat variable results regarding MDM4 rs4245739 and cancer risk may also be explained by yet unknown functional SNP (s) that are in linkage disequilibrium with rs4245739 [20].

This is the first meta-analysis to evaluate whether SNPs in the MDM4 gene are associated with cancer risk. We screened more than 500 titles and abstracts, and found that the relevant literature is focused on 5 SNPs (rs11801299, rs1380576, rs10900598, rs1563828, and rs4245739), and especially rs4245739. The reported research has involved various types of cancer, including ovaries, lung, stomach and other cancers. When performing meta-analyses, it is strongly recommended to investigate the influence of potential heterogeneity factors in order to avoid drawing simplistic and potentially misleading conclusions [31]. We therefore performed meta-regression, sensitivity analysis, and subgroup analysis in the present study. The main finding of the meta-analysis is that rs4245739 is significantly associated with a decreased cancer risk in the allelic, heterozygous, and dominant models. Due to the presence of significant heterogeneity, we conducted meta-regression by publication year, ethnicity, cancer type, genotyping method, and source of controls; the results highlighted ethnicity and genotyping method as a major driver of heterogeneity. Accordingly, subgroup analyses were performed based on ethnicity, and significant associations were only found in Asian populations. We also performed a sensitivity analysis to evaluate the robustness of the results, which revealed that no single study substantially changed the corresponding pooled ORs and 95% CIs. Five indices of publication bias were examined: the funnel plots and Begg's and Egger's linear regression tests indicated significant publication bias; however, using the trim-and-fill analysis correction results did not change, and the fail-safe number was larger than the number of included studies, which suggests that the positive results were robust despite the existence of publication bias.

Some limitations of this meta-analysis should be considered when interpreting its findings. Firstly, although we applied a highly sensitive search strategy to retrieve potentially eligible studies, we cannot rule out the possibility that some relevant studies were overlooked. Secondly, the number of eligible studies for the analyzed SNPs was small, which may have resulted in the statistical power being insufficient to detect weak but significant associations. Thirdly, most of the studies included in this meta-analysis involved Caucasian and Asian populations, and so further studies involving other ethnic populations are required. Finally, this study was a meta-analysis with a case–control design, and so the presence of confounding should be considered.

In summary, this meta-analysis has demonstrated that the MDM4 rs4542739 polymorphism was associated with decreased cancer risk, especially in Asian populations. However, due to the limitations listed above, the findings of this investigation should be interpreted with caution. Well-designed, multicenter, and large-cohort studies are needed to confirm our findings in the future.

## MATERIALS AND METHODS

This meta-analysis was performed according to the guidelines described in the PRISMA (Preferred Reporting Items for Systematic Reviews and Meta-analyses) statement [32] (PRISMA Checklist see Supplementary Table S1).

### Search strategy and study selection

To identify all published studies related to the relationships between MDM4 polymorphisms and cancer risk, we searched the following databases up to June 23, 2016: PubMed, Embase, China National Knowledge Infrastructure (CNKI, http://www.cnki.net/), and Wanfang Data (WD, https://www.wanfangdata.net/). The following MeSH (Medical Subject Heading) terms and/or text words were used in PubMed: “MDM4 protein, human,” “polymorphism, single nucleotide,” “genotype,” “mutation,” “alleles,” “genetic variation,” “neoplasms,” and “carcinoma.” The following Emtree terms were also used in Embase: “protein mdmx,” “genetic polymorphism,” “single nucleotide polymorphism,” “genotype phenotype correlation,” “mutation,” “allele,” “genetic variability,” “neoplasm,” and “carcinoma” (The full search strings see Supplementary Methods). We searched the CNKI and WD databases using the Chinese characters corresponding to these keywords. The reference lists in articles retained for review were also examined manually to further identify potentially relevant reports.

All of the studies included in the current analysis needed to meet the following criteria: (i) involved an assessment of the relationship between MDM4 polymorphism and cancer risk, (ii) had a case–control design, and (iii) provided sufficient information to estimate OR and 95% CI values.

### Data extraction

The following information about each study was extracted: first author's name, publication year, race, genotyping methods, cancer type, numbers of cases and controls, source of controls, and *P value* for HWE in controls. Publications were classified as involving different studies if they contained subjects with different cancer types or populations. All SNPs included in the subsequent meta-analysis were represented using dbSNP identifiers (i.e., rs numbers). For papers that did not report genotype or allele distributions, we sought the genotype information by directly e-mailing the first or corresponding author.

Data extraction was performed independently by two of the authors (Y.J.Z. and Z.J.D.), with any disagreements resolved by consensus.

### Quality score assessment

Two independent investigators (Y.J.Z. and Z.J.D.) assessed the quality of eligible studies using quality scoring criteria modified from those used in previous meta-analyses (Supplementary Table S4) [33, 34]. These modified criteria were based on traditional quality scoring protocols used for observational studies involving genetic epidemiology, and the scores ranged from 0 points (worst quality) to 15 points (best quality) [35]. The studies were then dichotomized into those of low quality (score < 10) and high quality (score ≥ 10).

### Statistical analysis

The associations between MDM4 polymorphisms and the risk of cancer were measured by ORs with 95% CIs based on five genetic models: allelic model, homozygous model, heterozygous model, dominant model, and recessive model. Statistical heterogeneity across studies was assessed using Cochran's *Q* test and *I*^2^ statistics, for which *P* < 0.1 and/or *I*^2^ > 50% indicated the presence of significant heterogeneity. The DerSimonian and Laird random-effects methods were used to calculate the OR if significant heterogeneity was present; otherwise the Mantel-Haenszel fixed-effects model was applied. Meta-regression analysis was undertaken to explore potential sources of heterogeneity across studies when statistical heterogeneity was detected. The covariates included publication year, ethnicity, cancer type, genotyping methods, and source of controls. In order to avoid false-positive results, the Bonferroni method was used to adjust the significance level of each covariate. Subgroup analyses were performed based on ethnicity cancer type, and source of controls.

Leave-one-out sensitivity analysis was conducted by sequentially excluding individual studies one at a time and recalculating ORs to evaluate the stability of the results. We also performed a sensitivity analysis by excluding the low-quality studies and computing the ORs for high-quality studies only. Publication bias was assessed using the funnel plot, Begg's and Egger's linear regression test, and the trim-and-fill method [36]. The fail-safe number of negative studies that would be required to nullify the effect size (i.e., to make *P* > 0.05) was also calculated [37].

A *P value* of < 0.05 (two-sided) was considered statistically significant. All statistical analyses were performed using Stata software (version 12.0, Stata Corporation, College Station, TX).

## SUPPLEMENTARY MATERIALS TABLES






