# Supplementary material for: A PRISMA-compliant meta-analysis of MDM4 genetic variants and cancer susceptibility
Source: Oncotarget. 2016 Oct 11;7(45):73935–44. doi: 10.18632/oncotarget.12558 (PMC5342025; doi:10.18632/oncotarget.12558)
Supplement: Supplementary file 1 [file oncotarget-07-73935-s001.pdf]

A PRISMA-compliant meta-analysis of MDM4 genetic variants and cancer susceptibility

Supplementary Materials

SUPPLEMENTARY METHODS: SEARCH CRITERION

1. PubMed Search String (MeSH Search)

(1) Polymorphism, Genetic [Mesh]; (2) Polymorphism, Single Nucleotide [Mesh]; (3) Genotype [Mesh]; (4) Mutation [Mesh]; (5) Alleles [Mesh]; (6) Genetic Variation [Mesh]; (7) 1 OR 2 OR 3 OR 4 OR 5 OR 6; (8) MDM4 protein, human [Supplementary Concept]; (9) Neoplasms [Mesh]; (10) Carcinoma [Mesh]; (11) 9 OR 10; (12) 7 AND 8 AND 11

2. PubMed Search String (Text Word Search)

(1) polymorphism; (2) single nucleotide polymorphism; (3) genotype; (4) mutation; (5) alleles; (6) variation; (7) 1 OR 2 OR 3 OR 4 OR 5 OR 6; (8) MDM4; (9) cancer; (10) tumor; (11) malignancy; (12) carcinoma; (13) neoplasm; (14) 9 OR 10 OR 11 OR 12 OR 13; (15) 7 AND 8 AND 14

3. Embase Search String (Emtree Search)

(1) 'protein mdmx'/exp; (2) 'genetic polymorphism'/exp; (3) 'single nucleotide polymorphism'/exp; (4) 'genotype phenotype correlation'/exp; (5) 'mutation'/exp; (6) 'allele'/exp; (7) 'genetic variability'/exp; (8) 2 OR 3 OR 4 OR 5 OR 6 OR 7; (9) 'neoplasm'/exp; (10) 'carcinoma'/exp; (11) 9 OR 10; (12) 1 AND 8 AND 11

4. Embase Search String (Quick Search)

(1) polymorphism; (2) single nucleotide polymorphism; (3) genotype; (4) mutation; (5) alleles; (6) variation; (7) 1 OR 2 OR 3 OR 4 OR 5 OR 6; (8) MDM4; (9) cancer; (10) tumor; (11) malignancy; (12) carcinoma; (13) neoplasm; (14) 9 OR 10 OR 11 OR 12 OR 13; (15) 7 AND 8 AND 14.

Supplementary Table S1: PRISMA 2009 Checklist For this meta-analysis. See Supplementary_Table_S1

Supplementary Table S2: The genotype data of included studies

SNP	Number of Genotype (Case)			Number of Genotype (Control)			Data source
	Wild type	Hetero-zygote	Homo-zygote	Wild type	Hetero-zygote	Homo-zygote	
rs4245739 A/C	86	61	7	88	56	9	Wynendaele 2010
	3318	2637	557	22825	15798	2828	Garcia 2013
	733	67	0	686	111	3	Liu (Jinan) 2013
	278	22	0	501	96	3	Liu (Huaian) 2013
	501	37	2	478	70	2	Zhou (Jinan) 2013
	529	56	3	510	88	2	Zhou (Huaian) 2013
	187	13	0	346	53	1	Fan 2014
	208	209	51	210	219	64	Feng 2014
	966	643	108	1021	703	146	Gansmo 2015
	823	600	108	2042	1439	266	Gansmo 2015
	715	515	101	2042	1439	266	Gansmo 2015
	1412	927	161	1021	763	120	Gansmo 2015
	297	22	1	548	90	2	Gao (Jinan) 2015
	183	17	0	321	77	2	Gao (Huaian) 2015
	716	564	105	1021	703	146	Gansmo 2016
	757	541	106	1021	703	146	Gansmo 2016
rs11801299 G/A	684	351	40	665	376	38	Yu 2011
	118	179	74	202	109	10	Yu 2012
rs1380576 C/G	487	477	111	518	455	106	Yu 2011
	170	158	43	150	135	36	Yu 2012
	188	281	173	212	290	218	Wu 2015
rs10900598 G/T	307	545	223	296	552	231	Yu 2011
	233	126	12	93	156	72	Yu 2012
rs1563828 C/T	53	57	14	44	43	14	Song 2012
	98	91	21	90	88	22	Zhang 2012
	27	21	2	389	340	70	Gansmo 2015

Supplementary Table S3: The result of sensitivity analysis for various genotype models. See Supplementary_Table_S3

Supplementary Table S4: Score of quality assessment

Criteria	Score
Representativeness of cases	
Selected from population cancer registry	2
Selected from hospital	1
Not described	0
Source of controls	
Population-based	2
Hospital-based	1
Not described	0
Ascertainment of cancer cases	
Histopathologic confirmation	2
Patient medical records	1
Not described	0
Control selection	
Controls matched with cases by age and sex	2
Controls matched with cases only by age or by sex	1
Not matched or not described	0
Genotyping examination	
Genotyping done blindly and quality control	2
Only genotyping done blindly or quality control	1
Unblinded and without quality control	0
Hardy-Weinberg equilibrium in controls	
Hardy-Weinberg equilibrium	2
Hardy-Weinberg disequilibrium or not described	0
Total sample size	
> 1000	3
501–1000	2
201–500	1
≤ 200	0
