# Supplementary material for: A PRISMA-compliant meta-analysis of MDM4 genetic variants and cancer susceptibility
Source: Oncotarget. 2016 Oct 11;7(45):73935–44. doi: 10.18632/oncotarget.12558 (PMC5342025; doi:10.18632/oncotarget.12558)
Supplement: Supplementary file 3 [file oncotarget-07-73935-s003.docx]

**Supplementary Table S3:** The result of sensitivity analysis for various genotype models

| SNP | Variables | No. of studies | Sample size (case/control) | Allele | | Homozygous | | Heterozygous | | Dominant | | Recessive | |
| --- | --- | --- | --- | --- | --- | --- | --- | --- | --- | --- | --- | --- | --- |
|  |  |  |  | OR (95%CI) | *P^h^/I^2^* | OR (95%CI) | *P^h^/I^2^* | OR (95%CI) | *P^h^/I^2^* | OR (95%CI) | *P^h^/I^2^* | OR (95%CI) | P^h^/I^2^ |
| rs4245739 A/C | All | 15 | 19796/49760 | 0.840  (0.755-0.935) | <0.001/88.0% | 1.005  (0.866-1.166) | 0.006  /54.8% | 0.819  (0.722-0.930) | <0.001  /86.0% | 0.813  (0.715-0.924) | <0.001  /87.6% | 1.006  (0.889-1.139) | 0.044/42.0% |
|  | Asian | 8 | 3416/4483 | 0.561  (0.439-0.718) | 0.001/71.1% | 0.782  (0.536-1.141) | 0.910/0.0% | 0.547  (0.428-0.698) | 0.007  /64.0% | 0.544  (0.428-0.692) | 0.007  /64.1% | 0.806  (0.562-1.156) | 0.918/0.0% |
|  | Caucasian | 7 | 16200/45277 | 1.022  (0.946-1.104) | <0.001/81.6% | 1.034  (0.876-1.219) | <0.001/75.2% | 1.034  (0.955-1.120) | 0.003  /69.4% | 1.032  (0.944-1.128) | <0.001  /78.0% | 1.024  (0.890-1.179) | 0.005/67.4% |
| rs11801299 G/A | All  (Caucasian) | 3 | 1766/1721 | 1.715  (0.531-5.545) | <0.001/98.5% | 3.549  (0.302-41.765) | <0.001/97.2% | 1.583  (0.523-4.794) | <0.001  /97.1% | 1.522  (0.672-3.448) | <0.001  /96.5% | 2.817  (0.401-19.803) | <0.001  /95.6% |
| rs1380576 C/G | All | 4 | 2408/2441 | 1.018  (0.931-1.114) | 0.393  /0.0% | 1.002  (0.831-1.208) | 0.558  /0.0% | 1.094  (0.958-1.250) | 0.918  /0.0% | 1.074  (0.957-1.206) | 0.909  /0.0% | 0.943  (0.797-1.116) | 0.461  /0.0% |
|  | Asian | 1 | 642/720 | 0.939  (0.807-1.091) | _ | 0.895  (0.676-1.184) | _ | 1.093  (0.846-1.411) | _ | 1.008  (0.798-1.273) | _ | 0.849  (0.671-1.075) | _ |
|  | Caucasian | 3 | 1766/1721 | 1.065  (0.953-1.191) | 0.735  /0.0% | 1.098  (0.853-1.413) | 0.850  /0.0% | 1.095  (0.937-1.279) | 0.680  /0.0% | 1.096  (0.960-1.253) | 0.919  /0.0% | 1.052  (0.827-1.339) | 0.948  /0.0% |
| rs10900598 G/T | All (Caucasian) | 3 | 1766/1721 | 0.530  (0.163-1.729) | <0.001/98.7% | 0.253  (0.019-3.363) | <0.001/98.2% | 0.559  (0.193-1.615) | <0.001  /96.6% | 0.576  (0.253-1.312) | <0.001  /96.2% | 0.341  (0.043-2.713) | <0.001  /97.4% |
| rs1563828 C/T | All | 2 | 260/999 | 0.889  (0.695-1.138) | 0.493  /0.0% | 0.740  (0.411-1.335) | 0.350  /0.0% | 0.930  (0.664-1.301) | 0.859  /0.0% | 0.893  (0.647-1.232) | 0.680  /0.0% | 0.768  (0.437-1.352) | 0.358  /0.0% |

*P^h^* value< 0.1 and/ or *I^2^* >50% showes the presence of significant heterogeneity.
